# The Second Wave of SARS-CoV-2 Circulation—Antibody Detection in the Domestic Cat Population in Germany

**DOI:** 10.3390/v13061009

**Published:** 2021-05-27

**Authors:** Anna Michelitsch, Jacob Schön, Donata Hoffmann, Martin Beer, Kerstin Wernike

**Affiliations:** Institute of Diagnostic Virology, Friedrich-Loeffler-Institut, Südufer 10, Insel Riems, 17493 Greifswald, Germany; anna.michelitsch@fli.de (A.M.); jacob.schoen@fli.de (J.S.); donata.hoffmann@fli.de (D.H.)

**Keywords:** SARS-CoV-2, domestic cat, seroprevalence, COVID-19, diagnostics, serology, felines, Germany

## Abstract

Registered cases of severe acute respiratory syndrome coronavirus 2 (SARS-CoV-2) infections in the German human population increased rapidly during the second wave of the SARS-CoV-2 pandemic in winter 2020/21. Since domestic cats are susceptible to SARS-CoV-2, the occurrence of trans-species transmission needs to be monitored. A previous serosurvey during the first wave of the pandemic detected antibodies against SARS-CoV-2 in 0.65% of feline serum samples that were randomly sampled across Germany. In the here-presented follow-up study that was conducted from September 2020 to February 2021, the seroprevalence rose to 1.36% (16/1173). This doubling of the seroprevalence in cats is in line with the rise of reported cases in the human population and indicates a continuous occurrence of trans-species transmission from infected owners to their cats.

## 1. Introduction

From the first detected cases of infection in late 2019 [1], the severe acute respiratory syndrome coronavirus 2 (SARS-CoV-2) spread around the world in a pandemic event [2]. In Germany, cases of human SARS-CoV-2 infections are registered by the Robert-Koch Institute (RKI), which is the German federal government agency responsible for disease control and prevention. As in most European countries, the first wave of SARS-CoV-2 infections in the human population was detected from February to March 2020 [3]. In August of 2020, cases of SARS-CoV-2 infections started to rise again, hitting a high at the end of November, when the overall case reports had doubled within one month [4]. Strict measures concerning the social activity of citizens [5] led to a decline in registered infection until February 2021 [4]. During this so-called second wave between August 2020 and February 2021, the occurrence of variants of concern of SARS-CoV-2 [6], and the changes in transmissibility that came along [7], were reported.

Ever since the start of the pandemic, the role of cats in the transmission of SARS-CoV-2 was questioned [8]. The susceptibility of cats to a SARS-CoV-2 infection and the transmission of the virus to cohoused uninfected cats were shown in animal trials [9,10,11,12]. Further, first case reports of naturally infected cats [13,14,15,16,17,18,19,20] and serological surveys [21,22,23,24] proved the occurrence of natural SARS-CoV-2 infections in cats. A survey on serum samples from German domestic cats, conducted during the first wave of the pandemic, showed that trans-species transmission from infected humans to their cats happened on a regular, though infrequent, basis [25]. Since the higher number of human infections might also lead to a higher risk in trans-species transmission, the collection of serum samples from domestic cats from all over Germany during the second wave of the pandemic was continued.

## 2. Materials and Methods

From September 2020 to February 2021, serum samples were collected from domestic cats during the clinical examination from the attending veterinarian. They were sent to the Synlab company, located in Augsburg, Germany, for hematology testing, which kindly provided the superfluous sera for the presented study. The samples were chosen randomly. Neither the health status of the cats nor the SARS-CoV-2 infection status of the owners was known. The only data given was the postal code of the veterinary clinic that took the serum sample. Overall, 1173 serum samples were tested for antibodies against SARS-CoV-2 by an indirect ELISA against the receptor-binding domain (RBD). The indirect ELISA was already validated before [26]. Samples with a positive or borderline result were additionally tested in an indirect immunofluorescence assay (iIFA) in order to confirm the ELISA result. Only serum samples that were positive in both the ELISA and the iIFA test were regarded as positive for SARS-CoV-2 antibodies. In addition, samples with positive or borderline results in the ELISA were tested for neutralizing antibodies by the use of a virus neutralization test (VNT). Both tests were described before and are based on the usage of Vero E6 cells (L 0929, Collection of Cell Lines in Veterinary Medicine (CCLV), Friedrich-Loeffler-Institut, Greifswald-Insel Riems, Germany), and the 2019_nCoV Muc-IMB-1 SARS-CoV-2 isolate [25,27]. Further, cross-reactivity of the aforementioned tests with sera that contain antibodies against feline coronavirus (FCoV) was ruled out during the previous SARS-CoV-2 surveillance study on the German cat population [25].

## 3. Results and Discussion

**Figure 1:**
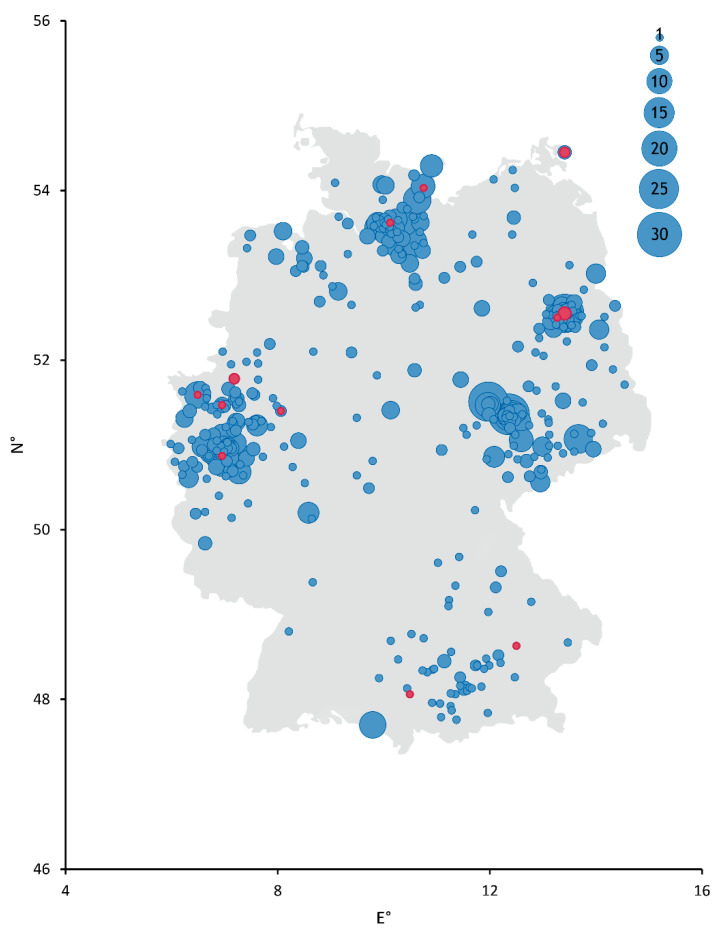
Schematic depiction of collection sites across Germany. Diameter of points correlates with the number of samples collected from each site. Serum samples that tested positive for antibodies against SARS-CoV-2 by ELISA and an indirect immunofluorescence test (iIFT) are colored red. Negative samples are colored blue. N° = decimal degrees of longitude and E° = decimal degrees of latitude.

The overall seroprevalence in the sampled cat sera during the second wave of human SARS-CoV-2 infections was 1.36% (16/1173) (see Table 1 and Figure 1), which is nearly twice as high as the prevalence during the first wave of virus circulation between April and September 2020 (0.65%) [25]. For comparison, the amount of registered human infections rose from 289,189 cases at the end of September 2020 to 2,442,336 at the end of February 2021, which accounts for a more than eightfold increase of overall registered human cases in Germany during the second wave of the pandemic [4].

In regard to the monthly detection rate, the highest rates were found in January (2.15%) and February (2.50%) 2021, occurring approximately one month after the direct virus detections in the human population had doubled within one month [4]. A similar effect was observed during the first wave, when the highest rate of ELISA positive feline serum samples was found approximately two months after the initial rise of SARS-CoV-2 infections in humans [25]. This delay is most likely due to the fact that antibody production starts not earlier than a week after the viral infection. However, studies on antibody kinetics in cats are still rare. An experimental study showed that sentinel cats acquiring a SARS-CoV-2 infection through inter-species transmission, which resembles an infection that might happen in a SARS-CoV-2 positive household, develop detectable levels of antibodies after 10 to 14 days [11].

On a federal state level, seroprevalences also corresponded to the number of infections detected in humans. In North Rhine-Westphalia and Bavaria, the highest rates of human infection in Germany were registered until the end of February, with percentages of 21.80% and 17.87% of all reported cases [4]. In accordance with that, antibodies against SARS-CoV-2 were detected in 2.03% (6/295) and 3.39% (2/59) of all collected feline serum samples originating from these federal states. However, the number of samples from each federal state varied; therefore, prevalences of these states have to be looked at with care. Nevertheless, the second-highest number of samples analyzed in the present study originated from Saxony, where only 7.95% of all human infections were registered until February 2021 [4]. In accordance, none of the 197 feline sera were found to be positive in the SARS-CoV-2 ELISA. In all 16 samples (100.00%) that tested positive by the RBD-ELISA antibodies were additionally detected in the iIFT against SARS-CoV-2. Neutralizing antibodies were found in 5 of the 16 positive serum samples (31.25%) by the VNT. These results confirm the findings of previous studies, in which neutralizing antibodies are found in only a small percentage of animals that were infected with SARS-CoV-2 [21,22,25]. A borderline result in the RBD-ELISA was detected in 9 of the 1173 serum samples (0.77%). Two of them (22.22%) were found positive in the iIFT against SARS-CoV-2 and none (0.00%) in the VNT (see Table A1). In a first follow-up study of two cats that acquired a SARS-CoV-2 infection from their owner, the level of detectable antibodies peaked at day 10 of sampling. Then, antibody levels started to decline and reached the limit of detection by ELISA after approximately four months [21]. Preliminary results of a second follow-up study of a naturally infected cat also indicate transient levels of detectable antibodies in domestic cats after infection with SARS-CoV-2. The examined cat was seropositive for two months, with a peak in detected antibody levels approximately one month after the start of the sampling [28]. Therefore, borderline samples are most likely originating from cats at an early stage of infection, in which case antibody production is only starting, or from cats that were infected earlier on, in which case antibodies are on a decline.

## 4. Conclusions

Overall, the conducted survey shows that trans-species transmission from infected humans to cats continues to happen on a regular basis. The doubling of the seroprevalence detected in the domestic cat population follows the rise in registered cases of human SARS-CoV-2 infections but not to a degree where a rise in the occurrence rate of trans-species transmission can be concluded.

## Figures and Tables

**Table 1:** Results of the indirect ELISA against the receptor-binding domain (RBD) of severe acute respiratory syndrome coronavirus 2 (SARS-CoV-2). The number of positive cat sera is given in the context of all tested samples for a specific month in each German federal state.

Federal State	Month 2020/21						
	September	October	November	December	January	February	Σ
Baden-Württemberg	0/0	0/0	0/2	0/1	0/0	0/0	0/3
Bavaria	0/19	0/1	2/27	0/7	0/5	0/0	2/59
Berlin	0/3	1/74	0/4	1/31	1/47	0/1	3/160
Brandenburg	0/0	0/17	0/3	1/13	0/19	0/15	1/67
Bremen	0/0	0/0	0/0	0/1	0/0	0/2	0/3
Hamburg	0/10	0/27	0/33	0/31	1/25	0/34	1/160
Hesse	0/7	0/3	0/3	0/1	0/2	0/0	0/16
Lower Saxony	0/2	0/12	0/6	0/12	0/18	0/7	0/57
Mecklenburg-Western Pomerania	0/1	0/0	0/0	1/1	0/1	1/14	2/17
North Rhine-Westphalia	2/63	1/112	0/13	0/56	3/51	0/0	6/295
Rhineland-Palatinate	0/4	0/4	0/0	0/0	0/0	0/0	0/8
Saarland	0/0	0/0	0/0	0/0	0/0	0/0	0/0
Saxony	0/35	0/38	0/40	0/40	0/44	0/0	0/197
Saxony-Anhalt	0/17	0/12	0/13	0/10	0/8	0/0	0/60
Schleswig-Holstein	0/1	0/13	0/14	0/16	0/12	1/7	1/63
Thuringia	0/2	0/2	0/1	0/2	0/1	0/0	0/8
Σ	2/164	2/315	2/159	3/222	5/233	2/80	16/1173

## Data Availability

Data is contained within the article.

## Appendix A

**Table A1:** Results of the indirect ELISA against the RBD of SARS-CoV-2, as well as of the virus neutralization test (VNT) and the indirect immunofluorescence assay (iIFT) against SARS-CoV-2.

Collection Date	RBD ELISA	iIFT SARS-CoV-2	VNT SARS-CoV-2
	Absorbance/Result		ND_100_
15 September 2020	0.799/positive	>1:1024	1:1024
23 September 2020	0.685/positive	>1:1024	neg. ^1^
6 October 2020	0.364/positive	>1:1024	1:101.6
28 October 2020	0.429/positive	1:512	neg. ^1^
10 November 2020	0.416/positive	>1:1024	neg. ^1^
11 November 2020	0.798/positive	1:64	neg. ^1^
7 December2020	0.588/positive	>1:1024	neg. ^1^
9 December 2020	0.822/positive	1:64	neg. ^1^
9 December 2020	0.364/positive	>1:1024	neg. ^1^
4 January 2021	0.354/positive	1:128	neg. ^1^
5 January 2021	0.837/positive	>1:1024	1:40.32
8 January 2021	0.709/positive	>1:1024	1:101.6
11 January 2021	0.425/positive	>1:1024	neg. ^1^
14 January 2021	0.827/positive	>1:1024	1:161.3
9 February 2021	1.108/positive	1:256	neg. ^1^
19 February 2021	0.539/positive	1:256	neg. ^1^
2 October 2020	0.264/borderline	neg. ^1^	neg. ^1^
21 October 2020	0.234/borderline	neg. ^1^	neg. ^1^
28 October 2020	0.208/borderline	neg. ^1^	neg. ^1^
2 November 2020	0.210/borderline	neg. ^1^	neg. ^1^
11 November 2020	0.265/borderline	1/128	neg. ^1^
29 December 2020	0.259/borderline	neg. ^1^	neg. ^1^
11 January 2021	0.215/borderline	neg. ^1^	neg. ^1^
22 January 2021	0.223/borderline	1:16	neg. ^1^
10 February 2021	0.212/borderline	neg. ^1^	neg. ^1^

^1^ neg. stands for a detection limit of <1:8 for the iIFT and <1:16 for VNT SARS-CoV-2.
